# Combination treatment with second generation antipsychotics other than clozapine

**DOI:** 10.1192/j.eurpsy.2021.108

**Published:** 2021-08-13

**Authors:** C. Schmidt-Kraepelin

**Affiliations:** Department Of Psychiatry And Psychotherapy, LVR-Clinic Düsseldorf - Medical Faculty Heinrich-Heine-University Düsseldorf, Düsseldorf, Germany

**Keywords:** Schizophrenia, Antipsychotics, Combination, Polypharmacy

## Abstract

**Background:**

Antipsychotic combination treatment without clozapine is common in the treatment of schizophrenia patients worldwide, despite clinical guidelines generally do not recommend such practice. This is potentially due to a high rate of non-response to monotherapies and a low-frequent adoption of Clozapine.

**Aim:**

This presentation briefly summarizes rational combination strategies without second generation antipsychotics other than clozapine and presents new results of a multi-center randomized, double-blind controlled trial comparing monotherapy of oral amisulpride (400-800 mg/day), or olanzapine (10-20 mg/day) with amisulpride-olanzapine combination treatment.

**Conclusions:**

Positive findings with small to medium effect sizes in favor of combination treatment with amisulpride and olanzapin have to be weight against a higher propensity to side effects since reduced sexual functions, weight gain and gain in waist circumference are higher in patients with combination treatment and olanzapine monotherapy than in patients with amisulpride monotherapy. Overall evidence in favor of combination treatment without clozapine is not strong when regarding its highly-frequent adoption in clinically practice. The strategy of combination treatment with amisulpride and olanzapine may be an alternative in certain clinical situations but should be carefully monitored and justified according to guideline recommendations for resistance to pharmacotherapy.

**Comments:**

The adoption of clozapine should be considered, before other antipsychotic combination treatment is indicated in clinical non-response to various monotherapies. Other factors that may lead to non-response or therapy resistance such as non-adherence, substance-abuse or high metabolization have to be excluded, before such strategy is appropiate.

**Disclosure:**

No significant relationships.

